# Occurrence of Aflatoxins in Selected Processed Foods from Pakistan

**DOI:** 10.3390/ijms13078324

**Published:** 2012-07-04

**Authors:** Muhammad Mushtaq, Bushra Sultana, Farooq Anwar, Muhammad Zargham Khan, Muhammad Ashrafuzzaman

**Affiliations:** 1 Department of Chemistry and Biochemistry, University of Agriculture, Faisalabad 38040, Pakistan; E-Mail: mmushtaq_doc@yahoo.com; 2 Department of Chemistry, University of Sargodha, Sargodha 40100, Pakistan; E-Mail: fqanwar@yahoo.com; 3 Department of Pathology, University of Agriculture, Faisalabad 38040, Pakistan; E-Mail: mzargham2000@yahoo.com; 4 Institute of Tropical Agriculture, Universiti Putra Malaysia (UPM), Serdang 43400, Selangor, Malaysia

**Keywords:** aflatoxins contamination, cereals based products, immunoaffinity clean-up, effective recovery, HPLC

## Abstract

A total of 125 (ready to eat) processed food samples (70 intended for infant and 55 for adult intake) belonging to 20 different food categories were analyzed for aflatoxins contamination using Reverse Phase High Performance Liquid Chromatography (RP-HPLC) with fluorescent detection. A solvent mixture of acetonitrile-water was used for the extraction followed by immunoaffinity clean-up to enhance sensitivity of the method. The limit of detection (LOD) (0.01–0.02 ng·g^−1^) and limit of quantification (LOQ) (0.02 ng·g^−1^) was established for aflatoxins based on signal to noise ratio of 3:1 and 10:1, respectively. Of the processed food samples tested, 38% were contaminated with four types of aflatoxins, *i.e.*, AFB1 (0.02–1.24 μg·kg^−1^), AFB2 (0.02–0.37 μg·kg^−1^), AFG1 (0.25–2.7 μg·kg^−1^) and AFG2 (0.21–1.3 μg·kg^−1^). In addition, the results showed that 21% of the processed foods intended for infants contained AFB1 levels higher than the European Union permissible limits (0.1 μg·kg^−1^), while all of those intended for adult consumption had aflatoxin contamination levels within the permitted limits.

## 1. Introduction

Aflatoxins are polyketide based potent liver carcinogenic, mutagenic and immunosuppressive compounds, primarily produced by food-borne fungi, mainly *Aspergillus* species such as *flavus*, *parasiticus*, *niger*, *nomius*, *pseudotamari* and *bombycids*, *etc*. These fungi can colonize a variety of products such as corn, maize, oilseeds, spices, groundnuts and tree nuts, *etc*., under favorable conditions, thus leading to food contamination and spoilage [1–4].

There are almost 20 different types of aflatoxins identified until now, among these B1, B2, G1 and G2 are more prominent while AFB1 is considered to be the most toxic [5]. The health issues related to aflatoxins are equally complex and demand more research. The ingested aflatoxin undergoes various possible pathways depending on different parameters like dose quantity, type of species, age, diet, and immune system of host. Exposure of biological systems to harmful levels of aflatoxin results in the formation of epoxide, which reacts with proteins and DNA leading to DNA-adducts, thus causing liver cancer [2,6].

Aflatoxins persist to some extent in food even after the inactivation of the fungi by food processing methods, such as ultra high temperature products, due to their significant chemical stability [7,8]. Infants are at much higher risks of health problems compared to adults [9]. The maximum legal limit allowed for AFB1 in infant food in the European Union is 0.1 μg kg^−1^ [10]. In developing countries, the majority of the people survive largely on cereal based diets. Consequently, nutritional deficiencies are very prevalent in populations consuming high levels of cereals, particularly in children [11]. Moreover, poor diet and multiple infectious hazards are associated with malnutrition and growth faltering in infancy and childhood [5].

Various approaches exist for the determination of aflatoxin in food and feed commodities. Generally, all analytical methods follow the basic protocol of extraction, clean-up, separation, detection, identification and quantification. However, the most widely used techniques are those which include a chromatographic step to separate the mycotoxin of interest like minicolumn chromatography, thin layer chromatography, high performance liquid chromatography and gas liquid chromatography. Although immunoassay-based quantitative methods are fast and promising, for mycotoxin research they have the possibility of producing misleading results because of cross-reaction and interference in the complex matrixes [12,13]. Therefore, a more selective treatment followed by specific purification is required before the analysis in such cases.

Some purification, preconcentration and clean-up protocols have been used over the years to enhance the sensitivity and selectivity of HPLC methods for the determination of aflatoxins in different food commodities. However most of such sample preparation techniques are tedious and offer less sensitivity [14–17].

Currently, immunoaffinity column (IAC) clean-up followed by RP-HPLC with fluorescence detector has emerged as a promising technique for the reliable detection and quantification of aflatoxins in diversified foods.

The aim of this study was, therefore, to provide information about aflatoxin levels in processed infant foods marketed in different regions of Pakistan by using IAC clean-up assisted RP-HPLC method with fluorescence detection.

## 2. Results and Discussion

### 2.1. Repeatability and Reproducibility

**Figure 1:**
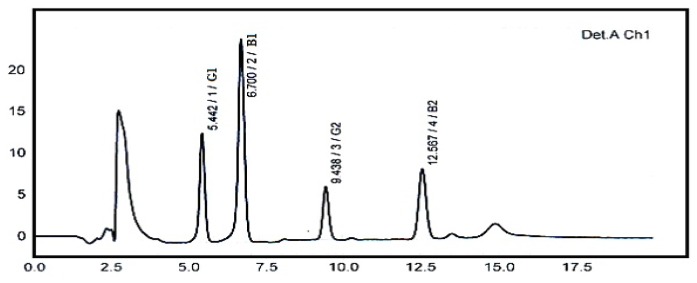
High Performance Liquid Chromatography (HPLC) Chromatogram of 5 ppb standard mixture of four aflatoxins.

A typical HPLC chromatogram showing the clear separation of 5 ppb standard mixture of four aflatoxins (AFB1, AFB2, AFG1 and AFG2) is depicted in Figure 1 below.

**Figure 2:**
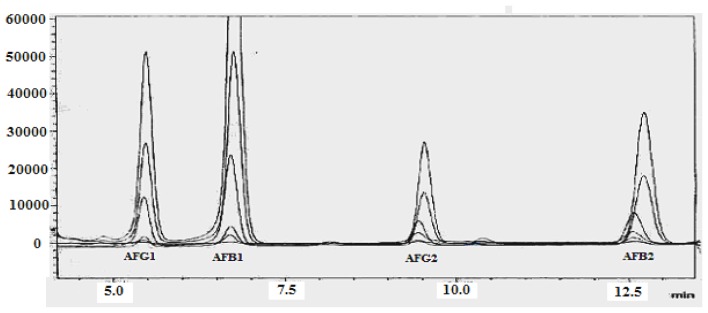
HPLC chromatogram showing the repeatability and reproducibility of AFGl, AFBl, AFGl and AFB2 standards.

As is evident from the HPLC chromatogram in Figure 2, the standard calibration curves were linear over 0.05–150 ng mL^−1^, 0.02–20 ng mL^−1^, 0.05–20 ng mL^−1^ and 0.02–6.0 ng mL^−1^ for AFB1, AFB2, AFG1 and AFG2, respectively, presenting a concentration dependent response and linearity of the detector.

The percentage recoveries were found to be 97.6% for AFB1 and AFG1 and 91.2% for AFB2 and AFG2 as shown in Table 1. A reasonably high recovery of the most important aflatoxin components (AFB1 and AFG1), as high as 97.6%, through spiking diversified foods, depicts that the method used is efficient and can be employed successfully for the reliable analysis of aflatoxins in processed food products. The present levels of aflatoxins percent recoveries were quite comparable with those earlier reported by Sizoo and Egmond [18].

### 2.2. Sample Analysis

**Figure 3:**
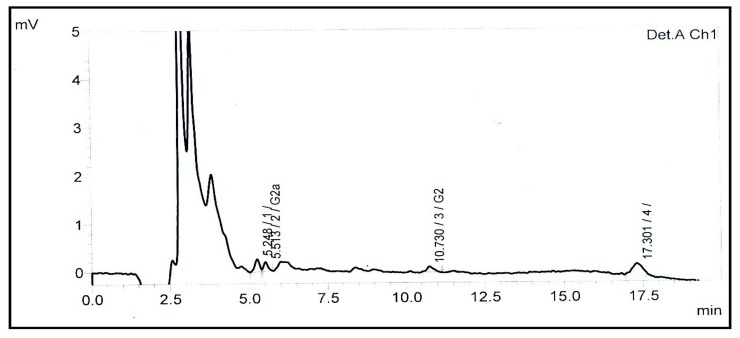
Typical chromatogram of uninfected wheat porridge processed food (sample shows undetectable amounts of aflatoxin).

The results obtained in this study showed variable levels of aflatoxin contamination in a variety of processed food collected from January–September, 2011. Overall, 37 % (47/125) of the processed food samples were found to be contaminated with aflatoxins. The incidence of alfatoxins in processed foods intended for infant use was 35% as shown in Table 2. The data showed that 21% (15/70) of the contaminated samples contained AFB1 higher than the permissible limits (0.01 μg kg^−1^) of the European Union (EC, 2006). The AFB1 and AFT level ranged 0.01–0.4 μg kg^−1^ and 0.02–3.8 μg kg^−1^, respectively. Individually 40% Cerelac, 33% Powder Milk, 50% Noodles, 20% Cream of rice, 20% Biscuits, 70% Corn Products, 20% Oatmeal, 20% Potato sticks and none of the Wheat Porridge samples (as shown in Figure 3) were found positive for aflatoxins contamination. These results depicted that difference in aflatoxins levels among the 20 different types of food were significant (*p* < 0.05).

There was no data available in the literature on the processed cerelac infant foods with which to compare the results of our present analysis.

### 2.3. Cerelac Infant Food Samples

Cerelac, a brand of cereal foods, is frequently used for infants and as a snack for the whole family in Pakistan as well as in several countries around the world. The brands are available in different flavors and composition depending upon the age and needs of the infant. During this study typically 20 cerelac food samples having different flavor and manufacturing dates were analyzed. The results obtained showed that aflatoxin contents of the foods varied depending upon their ingredients composition. On average 40% of the cerelac baby food samples were aflatoxin-contaminated, whereas rice and wheat-flavored products contained average AFB1 level (0.2 ± 0.01 μg kg^−1^) higher than the limits set by the European Union (EU).

### 2.4. Noodles

Noodles are used as food for children ranging between 1 and 6 years of age. This food product is usually derived from wheat, rice, legumes, or maize depending on their type and flavor [19]. Among the analyzed 10 noodle food samples, 5 (50%) were positive for aflatoxin contamination with amounts of 0.36 ± 0.01 μg kg^−1^ and (0.03 ± 0.01)–(0.40 ± 0.09) μg kg^−1^ for AFB1 and AFT, respectively. The reason for the high incidence of aflatoxin in noodles might be linked to the ingredients, especially, the corn flour [20]. The aflatoxin levels in 40% of the noodle samples were higher than European Union permissible limits (0.01 μg kg^−1^) and those reported by Sirhan *et al*. [21].

### 2.5. Baby Powder Milk

The US Federal Food, Drug, and Cosmetic Act (FFDCA) defines infant formula as “a food which purposes to be or is represented for special dietary use solely as a food for infants by reason of its simulation of human milk or its suitability as a complete or partial substitute for human milk”. So the composition of infant milk formula should be roughly based on a mother’s milk. The most commonly used infant formulas, as prescribed by manufacturers, contain purified protein, lactose, mixture of vitamins and minerals and other ingredients [22]. If these ingredients are obtained from cow’s milk, the infant powder milk might be contaminated with AFM1 instead of AFB1. However, the results showed the aflatoxin range of 0.17 ± 0.05 μg kg^−1^, 0.03 ± 0.01 μg kg^−1^, 0.06 μg kg^−1^, 0.11 ± 0.03 μg kg^−1^ and 0.07 ± 0.001 μg kg^−1^ for AFB1, AFB2, AFG1 and AFG2, respectively which are indications of the fact that baby powder milk samples were not mostly manufactured from cow’s milk.

Furthermore, the contamination level for AFB1 (0.2 ± 0.05 μg kg^−1^) was also higher than the limits set by the European Union. A large number of infants are fed with powdered milk around the world and likewise in Pakistan, so occurrence of AFB1 in milk samples can exert potential health hazards for infants in Pakistan, as infants are more susceptible to aflatoxin attack than adults [23,24].

### 2.6. Cream of Rice

Cream of rice is among the traditional foods of Pakistan and many other countries of the world. It is a type of paste that is somewhat thinner, made up of finely ground rice flour and often used in a sweet dish known as Kheer in Urdu and Hindi. Of the rice cream based products, 20% of the samples were found positive for the contamination of AFB1 and AFT at levels 0.07 ± 0.02 μg kg^−1^ and (0.03 ± 0.01)–(0.16 ± 0.05) μg kg^−1^, respectively, however these contents were within the safe limits as set by the European Union.

### 2.7. Biscuits

Sweet biscuits, commonly eaten as a snack food by children and adults, in general, are made with wheat flour, peanuts and oats, and sweetened with sugar or honey. There is usually a dedicated section for sweet biscuits in most Asian and European supermarkets. A variety of biscuits sold in Pakistan under different trade names were analyzed for their aflatoxin contamination. The trade name “Super biscuits”, having the ingredients wheat, milk and egg, as prescribed by the manufacturer, were found free of aflatoxins whereas the trade name “Good Man Biscuits” was found to have contamination level (0.31 ± 0.01 μg kg^−1^, 0.38 ± 0.01 μg kg^−1^, 1.13 ± 0.06 μg kg^−1^ and 0.68 ± 0.01 μg kg^−1^ of AFB1, AFB2, AFG1 and AFG2, respectively) as shown in Table 2.

### 2.8. Wheat Samples

Wheat is the most frequently used food commodity around the globe. A number of studies have already been carried out assessing the contamination of aflatoxin in wheat products. People in Pakistan use wheat flour from different sources e.g., commercial flour and home-made flour. In the present experiment, we selected three different types of Chapatties (bread). The first type was made from commercial wheat flour (the flour prepared commercially), second home-made wheat flour (produced from wheat washed with water and sun-dried), and third type prepared from air-cleaned wheat. The chapatti prepared with the air-cleaned wheat flour contained a higher level of AFB1 (0.28 μg kg^−1^) and total aflatoxin (1.2 ± 0.04 μg kg^−1^), followed by commercial wheat flour (1.3668 μg kg^−1^ of AFG1 and AFT) as shown in Table 3. The chapatti prepared from home-made wheat flour was noted to be free of aflatoxins. This showed that the aflatoxin contamination can be effectively reduced if the food commodities are washed with water before processing followed by sun-drying. The methods like roasting and extrusion processing show promise for lowering mycotoxin levels, but very high temperatures are needed to bring about effective reduction of such contaminants. According to a report, extrusion processing at temperatures greater than 150 °C is needed to achieve moderate reduction of alfatoxins [25]. Meanwhile, incorporation of washing and sun-drying steps in cereal processing can effectively remove aflatoxins. Furthermore, the levels of aflatoxins detected in our analysis were much lower than those of Giray *et al*. [26] who reported the range of aflatoxin contamination 10.5–65 μg kg^−1^ with incidence level 37%, compared to 55% found in our study and higher than Zinedine *et al*. [27].

### 2.9. Chines Fried Rice

Fried rice (common staple food in American and Asian Chinese cuisine) is made from steamed or pre-cooked rice with other ingredients such as eggs, vegetables and some kinds of meat. It is sometimes served as the penultimate dish in Chinese banquets. As a home-cooked dish, fried rice is typically made with ingredients left over from other dishes, leading to wider compositional variations. The results revealed the absence of AFB1 and AFB2 in all Chinese fried rice samples whereas AFG1 and AFG2 were detected at levels of 0.025 μg kg^−1^ and 1.3 μg kg^−1^, respectively. In contradiction to our present findings, in a recent study, Reiter *et al*. [28] analyzed a variety of unprocessed rice and found the presence of AFB1 (0.45–9.85 μg kg^−1^) where as AFG1 and AFG2 were not detected. Such variation might be attributed to the conversion of AFB1 and AFB1 into their corresponding derivative AFG1 and AFG2.

### 2.10. Milk Powder (Tea)

Powdered milk is a dairy product made by evaporating milk to dryness mainly for the purposes of long-term preserving and economic transportation. Many dairy products exported conform to standards laid out in Codex Alimentarius [29]. The incidence of aflatoxin contamination in tea milk powder collected from the local market manufactured by multinational companies was as high as 22%. Aflatoxins, namely AFB1, AFG1 and AFG2, with contributions of 0.05 μg kg^−1^, 0.15 μg kg^−1^ and 0.09 μg kg^−1^, respectively, were established in the tested powdered tea milk. Many studies have been conducted to investigating AFM1 (aflatoxin metabolite) in breast milk and animal milk samples [17,30–33] but none of these showed the presence of AFB1 in processed powder milk. The presence of aflatoxins, especially AFB1, in powder tea milk in the present analysis might be in part due to the fact that these products may be cereal–derived or the contamination may have been caused during the production.

### 2.11. Gram Flour

**Figure 4:**
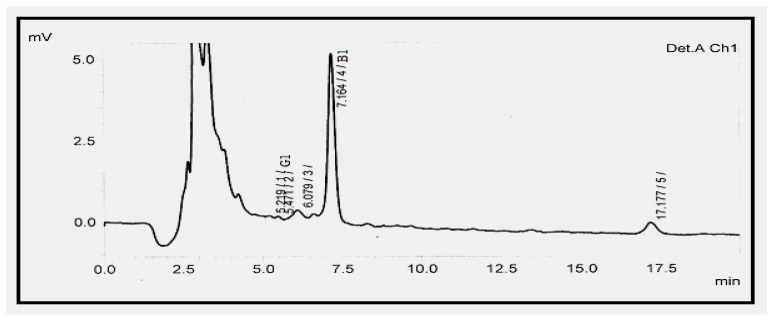
Chromatogram showing high level of AFB1 in gram flour.

Gram flour, also referred to as chickpea flour, garbanzo flour or besan, is used all over the world but as a staple in Indian, Pakistani and Bangladeshi cuisine. Gram flour is also commonly used as a paste with water or yoghurt to make a popular facial exfoliant in the Indian subcontinent and also popular in Pakistan as an ingredient in a number of different recipes including bonda, pakoras, papdums, onion bhajis and Bikaneri Bhujia. The aflatoxin level in gram flour was found to range from 0.32–1.02 μg kg^−1^ AFB1, 0.12 μg kg^−1^ AFB2, 0.59 μg kg^−1^ AFG1, 0.35 μg kg^−1^ AFG2 and 0.80 ± 0.06–2.1 ± 0.01 μg kg^−1^ AFT, respectively. The high amounts of aflatoxins in gram floor as depicted in Figure 4 might be due to insect related damage, high temperature and dry conditions during harvest and storage [33,34].

### 2.12. Barian

Barian, a local Pakistani food mostly used by the people in rural areas, is made by mixing meat, besan (gram flour), salt (which also acts as preservative) and spices followed by air and sun-drying. As is the case with gram floor (having 0.32–1.02 μg kg^−1^ AFB1), the ingredients composition of Barian supports that it may have high levels of aflatoxins. However, unexpectedly, the data analysis revealed the presence of aflatoxins at a comparatively lower concentration (0.07 μg kg^−1^ AFB1, 0.25 μg kg^−1^ AFG1, 0.15 μg kg^−1^ AFG2 and total aflatoxin (AFT) 0.03–0.48 ± 0.06 μg kg^−1^). These low levels of aflatoxins in this food product, compared with its basic ingredient (besan) might be due to sun-drying during the preparation of barian. We did not find any data on the detection of aflatoxins in barian with which to compare the present results.

### 2.13. Peanuts (Nimko)

**Figure 5:**
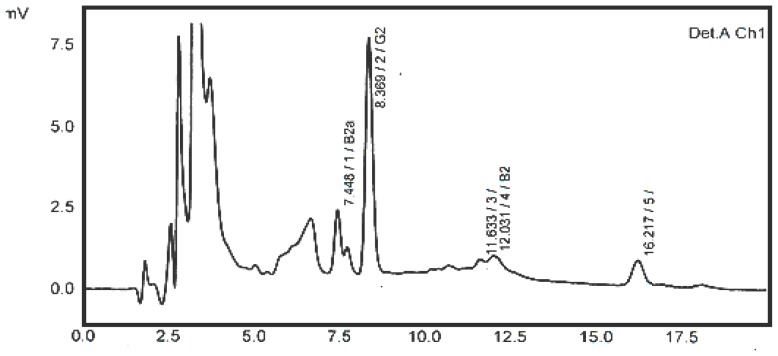
Typical HPLC chromatogram of aflatoxins in Nimko (peanut product).

Nimko or salto (local brand name), a ready to eat food taken by children as well as adults in Pakistan, mainly consists of roasted peanuts and spices. We analyzed five processed food samples of different brands collected from the local market and aflatoxin contamination is expressed by a typical chromatogram shown in Figure 5. As peanuts are among the cereals most susceptible for aflatoxin contaminations, therefore this food product (incidence level 60%) also showed the contamination of AFB1 at levels 0.2 μg Kg^−1^. This amount is even higher than EU regulated limits for ready to eat food for young children.

A number of studies have been reported on aflatoxin determination in nuts, showing high levels of contamination (9–71 μg Kg^−1^), but none of these studies dealt with the processed or mixed ready-to-eat nut products [35,36]. Furthermore, the lower amount of aflatoxins determined presently in Nimko (a peanut product) than that found in earlier studies might be the result of different steps such as frying, salting and extrusion involved during the production of Nimko.

## 3. Experimental Section

### 3.1. Collection of Samples

The samples of processed food products (*n* = 125), categorized as foods intended for infant (*n* = 70) and foods largely taken by adults (*n* = 55), were purchased from the local food stores of Lahore and Faisalabad region of Pakistan. The selected foods, derived from cereal grains, dairy and herbs, have been processed by the local and multinational manufacturers in Pakistan.

### 3.2. Chemicals and Materials

Aflatoxin standards were purchased from Supelco (Bellefonte, PA, USA). All other chemicals and reagents used were of analytical and HPLC grade from Merck (Darmstadt, Germany). The stock and working standard solutions were prepared in acetonitrile according to the Association of Official Analytical Chemists (AOAC) method [5] and stored at 20 °C in amber glass vials until analysis.

### 3.3. Extraction of Aflatoxins

Extraction of aflatoxins from food products was carried out by following the reported method of Beltran *et al.* [17] with slight modifications. Accurately weighed 5 g of representative sample was taken in a conical flask; mixed with 20 mL of extraction solvent (acetonitril:water 84:16) and shaken for 90 min in an orbital shaker at ambient conditions (average temperature 37 °C). The extract was filtered using Whatman filter paper No. 4 and the filtrate thus obtained was concentrated at 50 °C to a final volume of 2–5 mL by evaporation under reduced pressure.

### 3.4. Clean-Up

With the purpose to enhance the selectivity and sensitivity, 2–5 mL of concentrated sample was diluted with 20 mL of deionized water and passed through Vicam (waters) Aflatest WB immunoaffinity column at a flow rate of 2 mL min^−1^ with the help of suction pump. The immunoaffinity column was washed with a further 20 mL of deionized water and dried by air streaming for 1–2 min. The retained aflatoxins were eluted from the column by passing 2 mL of methanol in two steps (1 mL each). The samples thus obtained were dried under N_2_ blanketing.

### 3.5. Derivatization

Pre-column derivatization enhances the detection and recoveries of aflatoxin [16], which was done as follows:

200 μL *n*-hexane was added to the dried vial containing aflatoxin residues and vortexed for 30 s to remove the fat, then 50 μL of TFA (trifluro acetic acid) was added and the sample mixture vortexed again for 30 s followed by addition of 1.95 μL of water:acetonitril (9:1). The sample mixture was finally vortexed for 20 s and used for HPLC analysis.

HPLC Analysis Conditions: For quantitative estimation of aflatoxins, measurements were performed on LC-system in the Toxicopathological Laboratory, Department of Pathology, University of Agricultre, Fasialabad. An HPLC apparatus (Prominance^TM^, Shimadzu^®^, Kyoto, Japan) containing Shimadzu LC software package designed for HPLC real time and post operative analysis operated through computer equipped with Mediterranae Sea 18^®^ 5 μm; 25 × 0.46 cm Serial No. N45074 (Teknokroma, Barcelona, Spain) fitted with CTO-20A^®^ (Shimadzu, Kyoto, Japan) column oven and LC-20AT^®^ (Shimadzu, Kyoto, Japan) pump was used. Isocratic mobile phase consisting of acetonitrile, methanol and water ratio (22.5:22.5:55) was used at a flow rate of 1 mL min^−1^. Injection volume was 20 μL (Rheodyne^®^ sample injector with 20 μL sample loop). The elute was detected using spectrofluorometer detector RF-10A_XL_^®^ (Shimadzu, Kyoto, Japan) set at emission 440 nm and excitation at 360 nm. Limit of detection (LOD) was estimated as signal to noise ratio (S/N) = 3 and limit of quantification (LOQ) as (S/N) = 10.

### 3.6. Statistical Analysis

Triplicate samples were prepared and data thus obtained was analyzed statistically to calculate the level of significance of various parameters using analysis of variance technique by Minitab Software Package Version 13.0 (Minitab, Inc.: State College, PA, USA, 2000) and data were reported as mean ± SD. A probability level *p* < 0.05 was used to denote the statistically significant differences.

## 4. Conclusions

The results obtained in this study showed that the magnitude of AFB1 contamination varied widely among processed infant and adult foods. The levels of aflatoxins in the processed foods intended for infant consumption was found to be higher than the permissible limits set by the European Union. This can be more hazardous for infants, who are more sensitive and prone to exposure and toxic effects of such highly carcinogenic food contaminants. In addition, the amount of aflatoxins found presently was lower while the magnitude of their incidence was higher as compared with those reported for the unprocessed foods. This situation clearly demands wider national and international programs for the control of aflatoxin contamination in processed foods, especially in infant foods. The results of the present study may provide awareness regarding the aflatoxins in processed infant foods and adult food, from the point of view of food safety.

## Figures and Tables

**Table 1:** Repeatability, reproducibility and accuracy of HPLC method used for aflatoxin determination.

Aflatoxin	LOD a (ng g^−1^)	LOQ b (ng g^−1^)	Calibration Curve	*R*^2^	Recovery (%) c	Mean (μg kg^−1^) ± RSD (%) d
AFB1	0.02	0.05	y = 68,983x + 34,942	0.9997	97.6	125.3 ± 9.12
AFB2	0.01	0.02	y = 104,767x – 6,094	0.9995	91.2	15.3 ± 2.01
AFG1	0.02	0.05	y = 32,045x + 2,780	0.9996	97.6	15.3 ± 1.44
AFG2	0.01	0.02	y = 61,801x − 85.618	0.9991	91.2	6.3 ± 3.42

a Limit of detection;

b Limit of quantification;

c Accuracy was determined by the determination of the recoveries of aflatoxins. By spiking 125.5 μg kg^−1^ aflatoxin B1, 15.3 μg kg^−1^ aflatoxin G1 and B2 and 6.3 μg kg^−1^ G2 to the samples (uninfected ground and tree nuts);

d Replicate analysis of each spiked sample was used to determine the accuracy, expressed as mean (μg kg^−1^) ± relative standard deviation (%).

**Table 2:** Level of AFB1 and total aflatoxin (AFT) in processed food intended for infants.

Sample Typed	Number of Samples	Positive Samples	Samples Having AFB1 > 0.1 μg kg^−1^	Samples Having AFB1 < 0.1 μg kg^−1^	Total Aflatoxin (mean ± SD) mg kg^−1^
	*N*	*n* (%)	*n* (%)	*n* (%)	
Cerelac	10	4 (40)	2(20)	2 (20)	0.052 ± 0.020–0.19 ± 0.06
Powder Milk	10	3 (33)	2 (20)	1 (13)	0.030 ± 0.001–0.36 ± 0.07
Noodles	10	5 (50)	4(40)	1 (10)	0.025 ± 0.001–0.40 ± 0.09
Cream of rice	5	1 (20)	-	1(20)	0.025 ± 0.001–0.16 ± 0.05
Biscuits	10	3 (20)	1 (32)	2 (68)	(0.041 ± 0.02)–(2.48 ± 0.38)
Corn Products	10	7 (70)	5 (50)	2 (20)	(0.050 ± 0.020)–(3.74 ± 0.62)
Oatmeal	5	1 (20)	-	1 (20)	(0.02 ± 0.001)–(0.025 ± 0.001)
Potato sticks	5	1 (20)	1 (50)	1 (50)	(0.026 ± 0.001)–(2.96 ± 1.35)
Wheat Porridge	5	-	-	-	-

Total	70	25 (35)	15 (21)	10 (14)	-

**Table 3:** Incidence level and range of different types of aflatoxins in processed foods.

Sample Type	*n*	Incidence (%)	Aflatoxin Range (μg kg^−1^)				

			AFB1	AFB2	AFG1	AFG2	Total aflatoxin (AFT)
Chines fried rice	5	1 (20)	ND	ND	0.025	1.3	(0.03 ± 0.001)–(1.30 ± 0.002)
Bread slices	3	1 (33.3)	0.02–0.06	BD	0.06	ND	(0.1 ± 0.003)–(0.26 ± 0.004)
Sohn Halwa	3	1 (33.3)	BD	ND	ND	ND	(0.04 ± 0.001)–(0.16 ± 0.003)
Chapati local wheat cleaned with air	3	3 (100.0)	0.02–0.27	ND	ND	0.03–0.94	(0.05 ± 0.002)–(1.21 ± 0.04)
Chapati local wheat cleaned with water	3	0 (0)	ND	ND	ND	ND	ND
Chapati (commercial flour)	3	2 (66.7)	BD	0.025	1.36	0.03	(0.07 ± 0.003)–(1.36 ± 0.06)
Every Day Milk	9	2 (22)	0.0474	ND	0.141	0.084	(0.05 ± 0.002)–(0.27 ± 0.005)
gram flour	5	3 (60)	0.32–1.02	0.12	0.58	0.34	(0.8 ± 0.06)–(2.1 ± 0.01)
Peanuts (nimko)	5	3 (60)	0.21–1.24	0.13032	ND	1.741	(0.27 ± 0.003)–(2.08 ± 0.01)
Lays	3	1 (60)	BD	ND	0.57	ND	0.57
Bread slices	5	3 (20)	0.021	ND	0.06	ND	0.85
Soji Halwa	3	0 (0)	ND	ND	ND	ND	ND
Barian	5	1 (20)	0.07	0	0.25	0.15	(0.03 ± 0.001)–(0.48 ± 0.006)
Total	55	22 (40)					
